# Thymol Protects against 5-Fluorouracil-Induced Hepatotoxicity via the Regulation of the Akt/GSK-3β Pathway in In Vivo and In Silico Experimental Models

**DOI:** 10.3390/ph17081094

**Published:** 2024-08-21

**Authors:** Yasmen F. Mahran, Amira M. Badr, Layla A. Al-Kharashi, Hanaa N. Alajami, Nouf T. Aldamry, Nervana Moustafa Bayoumy, Elshaymaa I. Elmongy, Sahar Soliman

**Affiliations:** 1 Department of Pharmacology and Toxicology, Faculty of Pharmacy, Ain Shams University, Cairo 11566, Egypt; jassie_81@hotmail.com; 2 Department of Pharmacology and Toxicology, College of Pharmacy, King Saud University, Riyadh 11211, Saudi Arabia; lalkharashi@ksu.edu.sa (L.A.A.-K.); naldamri@ksu.edu.sa (N.T.A.); 3 College of Pharmacy, King Saud University, Riyadh 11211, Saudi Arabia; hanaalajmi16@gmail.com; 4 Department of Physiology, College of Medicine, King Saud University, Riyadh 11211, Saudi Arabia; nbayoumy@ksu.edu.sa; 5 Department of Pharmaceutical Chemistry, Faculty of Pharmacy, Helwan University, Ain Helwan, Cairo 11795, Egypt; shaymaa.taha@pharm.helwan.edu.eg; 6 Department of Physiology and Pharmacology, College of Osteopathic Medicine, Sam Houston State University, Conroe, TX 77304, USA; sahar.soliman@shsu.edu

**Keywords:** thymol, 5-flourouracil, hepatotoxicity, apoptosis, GSK-3β signaling, p44/42 MAPK(ERK1/2) signaling

## Abstract

Background: 5-fluorouracil (5-FU) is a widely used, highly effective chemotherapeutic agent. However, its therapeutic efficacy is often limited by associated adverse effects, with hepatotoxicity being frequently reported with 5-FU therapy. Thymol is a monoterpene found in thyme (*Thymus vulgaris* L., Lamiaceae) and is known for its antioxidant, anti-apoptotic, and anticancer activities. This study aimed to explore the hepatoprotective activity of thymol against 5-FU-induced liver injury. Methods: Rats received two intraperitoneal doses of 5-FU (150 mg/kg) either alone or in combination with thymol at doses of 60 mg/kg or 120 mg/kg. Liver enzymes, oxidative stress, and apoptotic markers, in addition to histopathological changes, were assessed. Results: 5-FU induced marked liver injuries as evidenced by elevated liver enzymes and histopathological changes, in addition to abnormalities of oxidative and apoptotic markers. The administration of thymol ameliorated the 5-FU-induced oxidative damage through increasing hepatic antioxidants and lowering lipid peroxidation. Apoptotic response markers such as Bax, Bcl-2, Bax/Bcl-2 ratio, and PARP were also improved. Furthermore, Western blotting analysis showed that thymol modulated the 5-FU-induced changes in the expression of Akt/GSK-3β and p44/42 MAPK (ERK1/2) signaling pathways. Conclusions: Our research is the first to shed light on thymol’s potential protective effect against 5-FU- induced hepatotoxicity by inhibiting oxidative and apoptotic pathways and modulating the Akt/ GSK-3β as well as p44/42 MAPK (ERK1/2) signaling pathways.

## 1. Introduction

Chemotherapy is a standard cancer treatment; however, its use can be limited by chemotherapy-induced hepatotoxicity. Efficient antineoplastic agents’ therapeutic outcomes are constrained because of the negative clinical side effects that necessitate dose reduction or a discontinuation of the therapy [1]. A fluorinated pyrimidine analog known as 5-fluorouracil (5-FU) is an antimetabolite chemotherapeutic agent extensively used for a variety of cancers, including breast and colorectal cancers [2]. 5-FU works by substituting the reduced thymidine triphosphate in DNA with the deoxyuridine triphosphate and fluorodeoxyuridine triphosphate, leading to DNA strand breaks [3]. 

Clinically, 5-FU treatment causes substantial systemic toxicities, including hepatotoxicity. Al-Asmari et al. (2016) reported the main mechanisms of 5-FU-induced cytotoxicity, including the overproduction of reactive oxygen species (ROS) and the generation of inflammatory mediators [4]. The use of 5-FU has occasionally been associated with the elevation of hepatic enzymes, such as alanine aminotransferase (ALT), aspartate aminotransferase (AST), and alkaline phosphatase (ALP). Moreover, 5-FU-generated oxidative stress in the liver causes lipid peroxidation and antioxidants depletion, leading to hepatocyte structural and functional abnormalities [5,6]. Thus, the use of antioxidants has been suggested to attenuate 5-FU-induced toxicity [7,8]. Finding approaches to reduce 5-FU-associated hepatotoxicity is crucial, as it may prove beneficial not only for minimizing liver damage but also for maintaining the optimal dosing required for effective chemotherapy, thereby improving the overall clinical outcomes of 5-FU [1]. 

As the search for effective ways to mitigate 5-FU-associated hepatotoxicity continues, the potential of natural substances, including essential oils from aromatic herbs, is being explored. These oils are known for their distinctive aromas, as well as their diverse therapeutic properties such as insecticidal, antibacterial, and antifungal activities [9]. Thymol, the primary component of the *Thymus vulgaris* component, is frequently used in foods to enhance flavor and taste due to its safety and acceptance [10,11]. In various experimental models, thymol is well known for its chemoprotective activity through antioxidant [12] and anti-inflammatory [13,14,15,16] mechanisms, due to its natural monoterpene phenolic composition [17], and has a favorable safety profile [18]. Thymol is also known to improve intestinal barrier integrity and fight off dangerous microbes, both of which contribute to its ability to support the immune system [19]. With regards to thymol’s hepatoprotective effect, thymol prevented the hepatic damage mediated by lipopolysaccharides via reducing inflammation and hepatocellular apoptosis [20]. Furthermore, thymol was used to ameliorate the ethanol- and nano titanium dioxide-induced liver injury through its antioxidant qualities [10,21]. However, the hepatoprotective mechanisms of thymol remain poorly understood. Understanding the different protective mechanisms is essential to identify the possible practical applications of thymol [20]. 

Glycogen synthase kinase-3 (GSK-3) is a ubiquitously expressed serine/threonine kinase with diverse functions in various cell types [22]. It is involved in a variety of cellular functions and regulates various transcription factors that define cell destiny. The role of the Akt/GSK-3β signaling pathway in acetaminophen-induced hepatotoxicity has been documented. Therefore, a detailed exploration of the GSK-3β signaling pathway in disease pathogenesis is warranted [23]. Several studies reported that thymol offered protective effects through the modulation of Akt/GSK-3β signaling in different experimental models [24,25]. Thus, we hypothesized that thymol can protect against 5-FU-induced liver injury by modulating oxidative and apoptotic-signaling molecules. Consequently, this study sought to investigate the potential hepatoprotective effect of thymol against 5-FU-induced liver injury, as well as the underlying protective mechanisms.

## 2. Results

### 2.1. Protective Effect of Thymol against 5-FU-Induced Histopathological Changes in Liver Tissues

**Figure 1:**
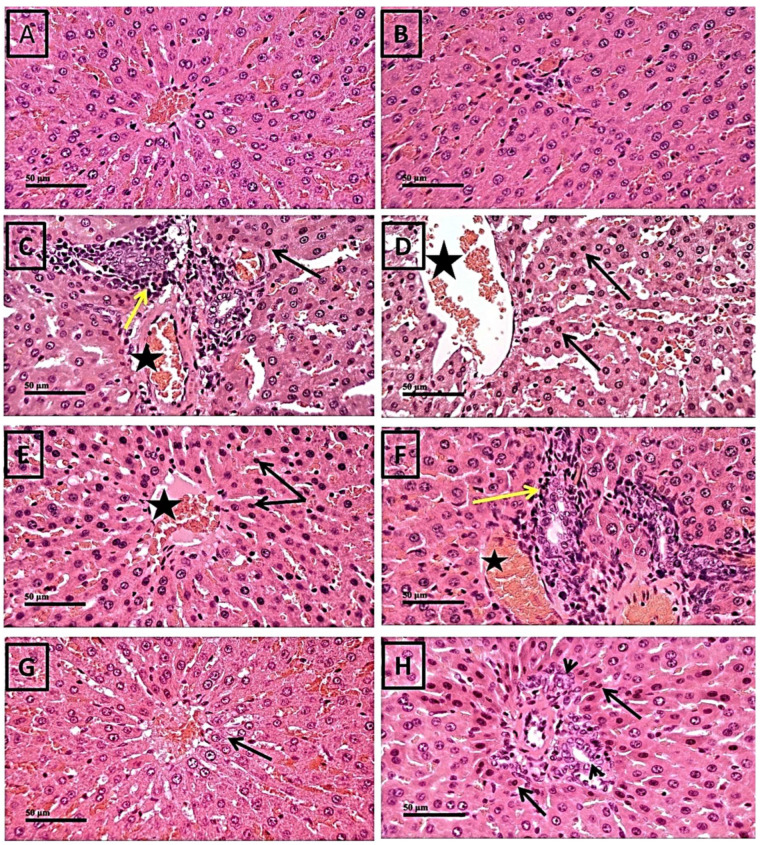
Histological photomicrographs of the liver tissue stained with hematoxylin and eosin staining. (**A**,**B**) Sections of liver tissues from the control rats received only the vehicle orally show normal histological features of hepatic parenchyma with normal hepatocytes. (**C**,**D**) Sections of liver tissues from the 5-FU treated rats that received the vehicle and two doses of 5-FU (150 mg/kg) show a dilatation of hepatic blood vessels (star) in both (**C**,**D**), periportal mononuclear inflammatory cell infiltrations (yellow arrow) in (**C**), and degenerated pyknotic hepatocytes in hepatic plats (arrow) in both (**C**,**D**). (**E**,**F**) Sections of liver tissues from the FU+ Thymol (60 mg) treated group that received thymol (60 mg/kg orally) and two doses of 5-FU (150 mg/kg) show more or less the same records as the 5-FU group with the same annotations. (**G**,**H**) Sections of liver tissues from the FU+ Thymol (120 mg) treated group that received thymol (120 mg/kg orally) and two doses of 5-FU (150 mg/kg) show intact hepatocytes with mild vacuolar changes in pericentral zones (arrow) as shown in (**G**). (**H**) shows few karyopyknotic changes in periportal areas (arrow) and minimal records of inflammatory cell infiltrates in periportal areas (arrowhead). X: 200 bar 200 μm, X: 400 bar 50 μm. FU: 5-fluorouracil.

The histopathological changes in the examined hepatic sections are shown in Figure 1. The control group revealed almost intact histological structures of hepatic parenchyma, with apparent normal hepatocytes showing large vesicular nuclei and intact vasculature. (Figure 1A,B). On the other hand, the administration of 5-FU demonstrated a moderate dilation of hepatic blood vessels (star) and sinusoids accompanied with periportal mononuclear inflammatory cells infiltrations (yellow arrow), scattered throughout the samples. Additionally, alternating intact or degenerated pyknotic hepatocytes in hepatic plates (arrow) were observed (Figure 1C,D). 

Concurrent treatment with 60 mg/kg thymol demonstrated a moderate protective effect against 5-FU-induced histopathological liver changes, yielding results comparable to those observed in the 5-FU group (Figure 1E,F). On the other hand, thymol administration (120 mg/kg) attenuated 5-FU-induced pathological changes, showing many apparent intact hepatocytes with mild vacuolar changes in pericentral zones (arrow) or few karyopyknotic changes in periportal areas (arrow). Minimal records of inflammatory cells infiltrating the periportal areas with mild hyperplasia of bile ducts (arrowhead) were observed (Figure 1G,H).

### 2.2. Effects of Thymol and 5-FU on Liver Enzymes and Oxidative Stress Markers in Liver Tissues

As shown in Table 1, the activities of liver enzymes AST, ALT, ALP, and LDH were markedly increased in the 5-FU treated group to about 142%, 188%, 238%, and 198%, respectively, compared to the control group. However, treatment with thymol (60 mg/kg) decreased serum levels of AST, ALT, ALP, and LDH by 15%, 46%, 61%, and 13%, respectively, compared to the 5-FU group, reflecting the protective effect of thymol. Furthermore, a significant reduction in liver enzymes was observed with thymol at a dose of 120 mg/kg by 35%, 65%, 70%, and 45%, respectively, when compared to the 5-FU group. The protective effect of thymol is dose-dependent, as shown in Table 1.

An increment in oxidative stress was evidenced in the 5-FU-treated group. The levels of SOD and GSH were reduced by 37% and 25%, respectively, compared to the control group. Additionally, the level of TBARSs was significantly elevated by approximately seven-fold in the 5-FU rats, reflecting increased lipid peroxidation. Conversely, the antioxidant effect of thymol was demonstrated by the increase in the level of GSH and SOD, reaching about 76% and 147%, respectively, and decreased the level of TBARSs by 35% at a dose of 60 mg/kg. 

On the other hand, the higher thymol dose (120 mg/kg) significantly increased the levels of SOD and GSH to about 270% and 144%, respectively, and the level of TBARSs was almost normalized compared to the control group.

Control; 5-FU rats; 5-FU + Thymol (60 mg/kg): 5-FU rats treated with thymol (60 mg/kg/day, orally); 5-FU + Thymol (120 mg/kg): 5-FU rats treated with thymol (120 mg/kg/day, orally).

x, y, or z: The 5-fluorouracil and 5-FU + thymol (60 mg/kg) groups are significantly different from the control at *p* < 0.05, using ANOVA followed by Tukey–Kramer as a post-hoc test. 5-FU: 5-fluorouracil; AST: aspartate aminotransferase; ALT: alanine aminotransferase; ALP: alkaline phosphatase; LDH: lactate dehydrogenase; SOD: superoxide dismutase; TBARSs: thiobarbituric acid reactive substances.

### 2.3. Effect of Thymol and 5-Fluorouracil on Apoptotic Marker Expression in Liver Tissues 

**Figure 2:**
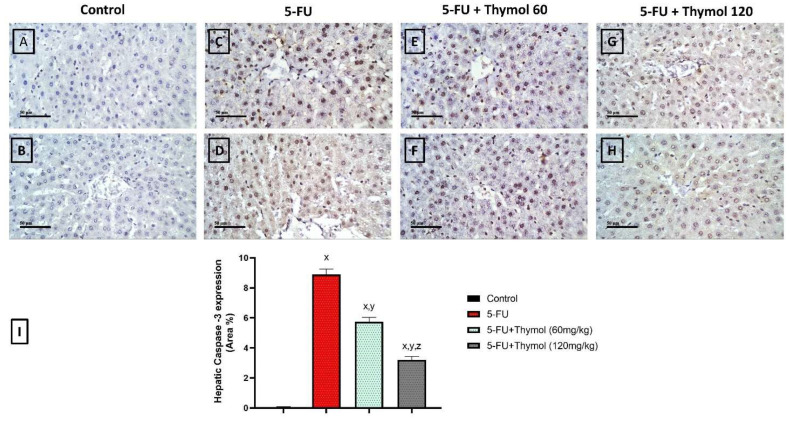
Effect of 5-fluorouracil with or without thymol on caspase-3 expression in liver tissues. (**A**–**H**) Immunohistochemical images of caspase-3 hepatic expression. (**A**,**B**) Control: normal rats that received only the vehicle orally show minimal caspase-3 expression. (**C**,**D**) FU rats: 5-FU treated rats that received the vehicle and two doses of 5-FU (150 mg/kg) show intense expression of caspase-3. (**E**,**F**) FU+Thymol 60: the FU+ Thymol (60 mg) treated group that received thymol (60 mg/kg orally) and two doses of 5-FU (150 mg/kg) show moderate caspase-3 expression. (**G**,**H**) FU+Thymol 120: the FU+ Thymol (120 mg) treated group that received thymol (120 mg/kg orally) and two doses of 5-FU (150 mg/kg) show moderate caspase-3 expression. (**I**) Statistical analysis of quantitative hepatic expression of caspase-3 as area % of immune-positive staining in all groups. Values are mean ± SEM (*n* = 5). x, y, or z: The 5-fluorouracil and Fu+ thymol (60 mg/kg) groups, are significantly different from the control at *p* < 0.05. *p* ≤ 0.05, as evaluated using ANOVA followed by Tukey–Kramer as a post-hoc test. Scale bar = 50 μm. 5-FU: 5-fluorouracil.

**Figure 3:**
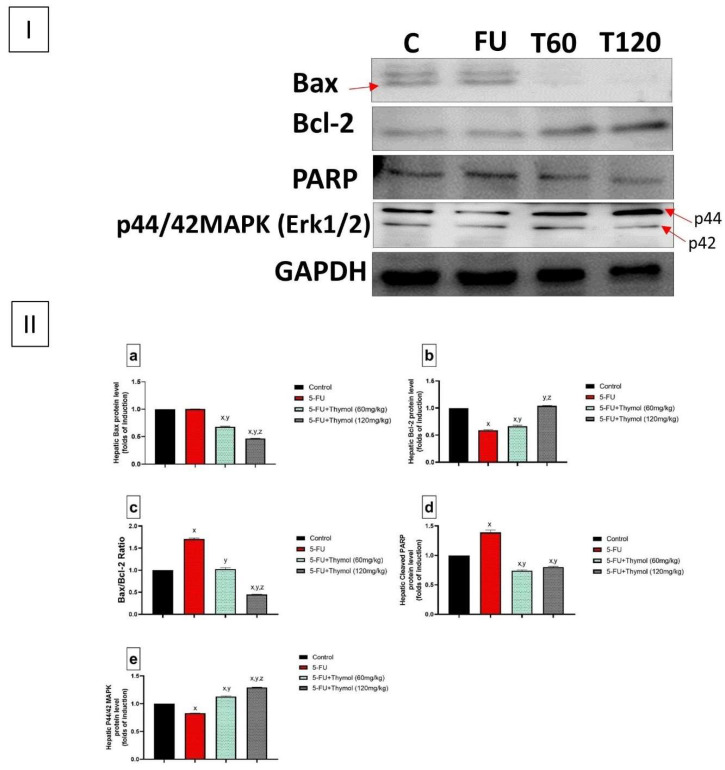
Effects of 5-fluorouracil with or without thymol on the hepatic protein expressions of the apoptotic markers Bax, Bcl-2, PARP, and p44/42 MAPK (ERK1/2) in rats. (**I**) Representative immunoblot of protein levels of Bax (lower band), Bcl-2, PARP, and p44/42 MAPK (ERK1/2) marked for p44 and p42 in liver tissues. (**II**) Quantitative results of the immunoblot of (**a**) Bax, (**b**) Bcl-2, (**c**) Bax/Bcl-2 ratio, (**d**) PARP, and (**e**) p44/42 MAPK (ERK1/2) protein levels are expressed as the ratio of expressed protein /GAPDH. The densities of immunoblots were quantified using analysis software. The relative quantities were normalized to the control and expressed as a fold of induction. Values are mean ± SEM (*n* = 3). x, y, or z: 5-Fluorouracil and 5-FU+ thymol (60 mg/kg) groups, are significantly different from the control at *p* < 0.05. *p* ≤ 0.05 was evaluated using ANOVA followed by Tukey–Kramer as a post-hoc test. 5-FU: 5-fluorouracil. p44/42 MAPK: p44/42 mitogen activated protein kinase.

To investigate the potential anti-apoptotic effects of thymol against 5-FU-induced hepatocellular death, the hepatic expression of caspase-3, the pro-apoptotic protein (Bax), and the anti-apoptotic protein (Bcl-2) were examined across the different groups. In addition, an assessment of the cleaved PARP (89 kDa) and p44/42 MAPK (ERK1/2) hepatic protein expression was also carried out. First, Figure 2 shows microscopic photos of caspase-3 expression in all experimental groups by immunohistochemical staining, where the control showed minimal caspase-3 expression while the FU-treated group showed an intense expression of caspase-3. However, thymol-treated groups show a moderate expression of caspase-3. A statistical analysis of area % of caspase-3 immunostaining revealed a significant induction of caspase-3 expression to about eight-fold in rats treated with 5-FU when compared to the control group. On the other hand, either dose of thymol (60 mg or 120 mg) significantly decreased caspase-3 expression to about 0.75- and 0.5-fold, respectively, compared with the 5-FU group, as shown in Figure 2. Moreover, the immunoblotting analysis showed that 5-FU increased Bax and decreased the Bcl-2 protein level by about 1- and 0.5-fold, respectively, relative to the control group (Figure 3a,b). Thus, the Bax/Bcl-2 ratio in the 5-FU group was significantly elevated to about 1.75 folds when compared to the control group. Nevertheless, treatment with thymol at a dose of 60 mg/kg significantly decreased the Bax/Bcl-2 ratio to a level comparable to that in the control group. Interestingly, the administration of thymol 120 mg/kg was able to decrease the Bax/Bcl-2 ratio to 0.4-fold compared to the control and the thymol 60 mg/kg group (Figure 3c). 

Next, we checked the effect on cleaved PARP and p44/42 MAPK (ERK1/2) hepatic protein expression. 5-FU strongly induced the expression of hepatic cleaved PARP (89 kDa) protein level by about 1.75-fold as compared to the control. Thymol (60 and 120 mg/kg) significantly decreased the hepatic cleaved PARP protein level below the level of the control group, reaching about 0.75-fold of the 5-FU group (Figure 3d). This shows that thymol might suppress 5-FU-induced liver damage through an inhibition of the apoptotic pathway. In addition, Figure 3e shows that 5-FU administration caused a 0.8-fold downregulation of the hepatic expression of p44/42 MAPK (ERK1/2) when compared to the control group, while thymol co-administration generated a significant p44/42 MAPK (ERK1/2) upregulation in a dose-dependent manner by 1.1-fold for thymol (60 mg/kg) and 1.3-fold for thymol (120 mg/kg), compared to both the control and the 5-FU values (Figure 3e).

### 2.4. Effect of Thymol and 5-Fluorouracil on Akt1/ GSK-3α/β Signaling Pathway Protein Expression in Liver Tissues

**Figure 4:**
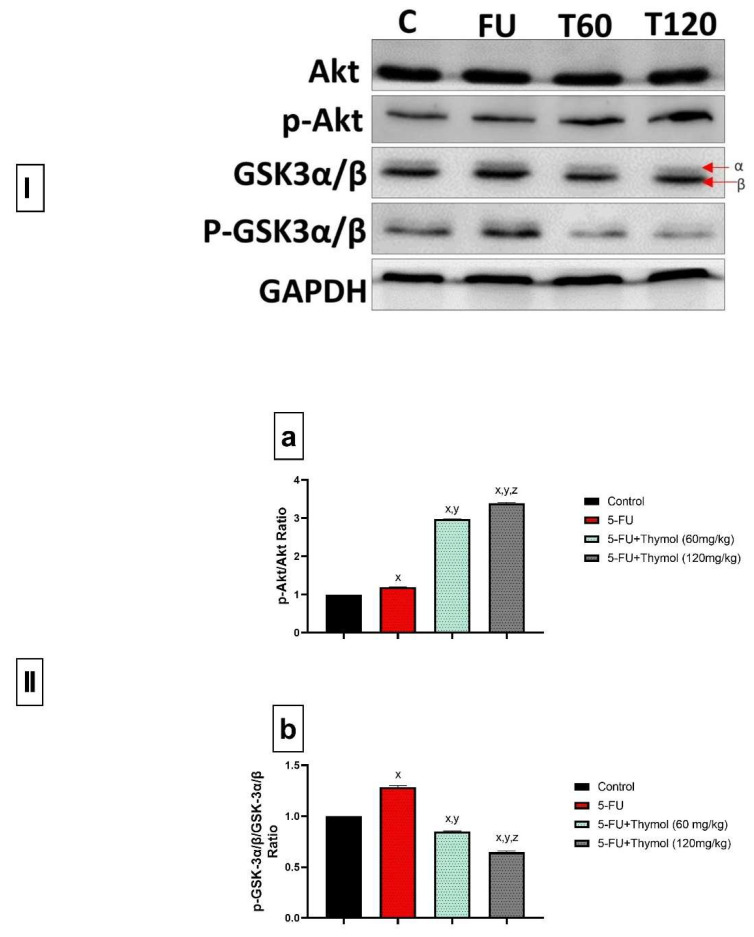
Effects of 5-fluorouracil with or without thymol on the p-AKT/p-GSK3α,3β signaling pathway in liver tissue of rats. (**I**) Representative immunoblot of protein levels of AKT, p-AKT, GSK3α,3β, and p-GSK3α,3β (the upper band for α and the lower band for β) in liver tissues. Proteins were immunoblotted with Akt antibody in “the first upper panel,” p-Akt antibody in “the second panel,” GSK3α,3β antibody in “the third panel,” p-GSK3α,3β antibody in “the fourth panel,” and after stripping with GAPDH antibody as a loading control in the “lower panel.” (**II**) Quantitative results for the immunoblot, showing (**a**) the ratio of p-Akt/Akt and (**b**) the ratio of p-GSK3α,3β/GSK3α,3β protein levels expressed, and the relative quantities were normalized to the control and expressed as a fold of induction. The densities of immunoblots were quantified using analysis software. Values are mean ± SEM (*n* = 3). x, y, or z: The 5-fluorouracil and Fu+ thymol (60 mg/kg) groups are significantly different at *p* < 0.05. *p* ≤ 0.05 was evaluated using ANOVA followed by Tukey–Kramer as a post-hoc test. 5-FU: 5-fluorouracil; Akt: protein Kinase B; p-Akt: phosphorylated Akt, GSK3α,3β: glycogen synthase kinase 3 α and 3β; p-GSK3α,3β: phosphorylated glycogen synthase kinase 3 α and 3β.

To further explore mechanisms of 5-FU-induced liver injury, we investigated the fundamental role of the AKT1/GSK-3α/β signaling pathway in the hepatic tissues. Results showed that 5-FU slightly upregulated the phosphorylation of both Akt1 and GSK-3α/β, which was evidenced by an increase in the ratio of p-Akt1/Akt1 and p-GSK-3α/β/GSK-3α/β by 1.2 and 1.3-folds, respectively, compared with the control group (Figure 4a,b). In this context, we found that the GSK-3α protein was less expressed in the hepatic tissue specimens among all different groups. Thus, the discussion will mainly take the GSK-3β isoform into consideration. However, the co-treatment of 5-FU-injected rats with thymol, either 60 or 120 mg/kg, modulated the Akt/GSK-3α/β signaling via an upregulation of the Akt1 phosphorylation downregulation of GSK-3α/β phosphorylation, compared to the 5-FU treated group, as shown in Figure 4a,b where the ratio of p-Akt1/Akt1 was increased about three-fold and the phosphorylation of GSK-3α/β/ decreased by about 40%.

### 2.5. Docking Studies of Thymol

**Figure 5:**
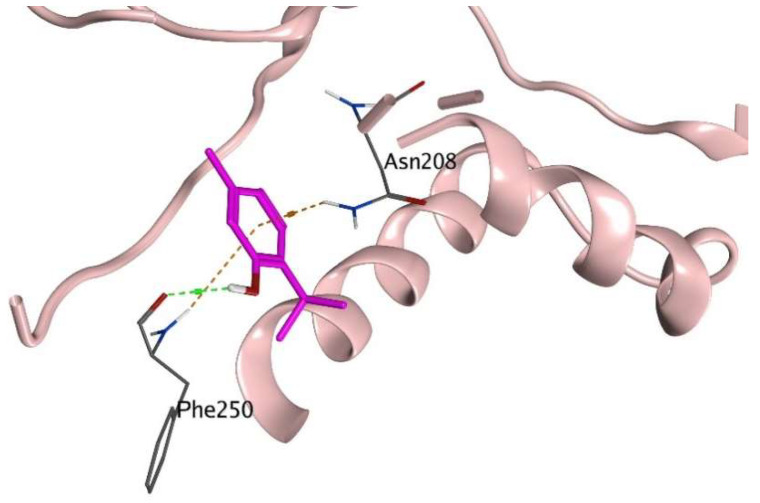
Thymol (in pink) interacting at the caspase-3 site of action with an H-bond as a green dotted line and pi-interactions as brown dotted lines.

This study investigates the protective effect of thymol against 5-FU-induced hepatoxicity, oxidative stress, and apoptosis. The counteraction of thymol to the aforementioned 5-FU-induced toxicities was also investigated computationally in addition to pharmacologic investigations on the docking of the markers’ downloaded proteins where pdbID:7JL7 represented the caspase-3 substrate, pdb ID:1Q5K represented the GSK-3β protein, and PDBID: 4S0P represented the BAX protein. As shown in Figure 5, thymol interaction with caspase-3 target protein involved PHE 250 and ASN 208 as amino acid residues in H-bond and hydrophobic interactions, respectively, and as ASP was not involved in the interactions, this might imply a decrease in caspase-3 levels, which are known to be aspartate specific [26].

**Figure 6:**
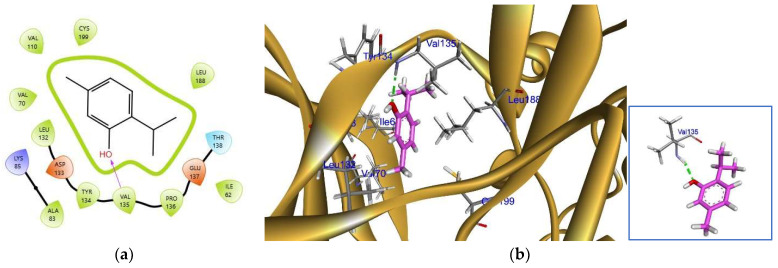
Thymol in GSK-3β: (**a**) 2D representation of thymol with a perfect fitting inside the active site and (**b**) 3D representation of thymol (in pink) interacting with valine 135 with a hydrogen bond (in green dotted line).

Docking on GSK-3β was performed based on reported inhibitors that showed activity against many diseases, such as cancer, Alzheimer’s disease, and chronic inflammatory diseases [27,28,29,30]. Results recorded a binding energy of −5.283 kcal/mol and were shown totally inside the binding site with a perfect fit of 1.082 Å and an interaction with VAL 135 by a hydrogen bond between the phenolic hydroxy group of thymol and the amino group of valine as shown in Figure 2. These results are consistent with those of the co-crystallized inhibitor “TMU901”, which interacts by a hydrogen bond with VAL 135 (Figure 6).

**Figure 7:**
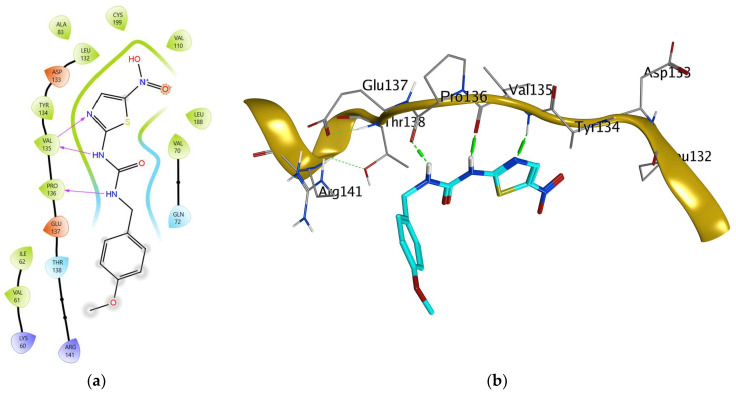
Co-crystalized ligand of GSK (displayed in blue colored sticks) in the binding site of GSK target protein (golden ribbon): (**a**) 2D presentation and (**b**) 3D visualization (site view) where hydrogen bonds are represented as dotted green lines.

Upon docking in the BAX (pdb: 4S0P) binding pocket, thymol recorded a binding energy of −4.595 kcal/mol and was totally in the site of interaction with a fitting at 1.487 Å but did not show any significant interaction, which is consistent with the biological results of reduced BAX level (Figure 7).

Table 2 presents the docking results for thymol with all of the downloaded proteins, detailing the types of chemical bonding, the amino acids involved in the interaction, and the recorded values for binding affinity and root mean square deviation (RMSD).

## 3. Discussion

It is undeniable that antineoplastic agents have been one of the major therapeutic modalities widely used for the eradication of cancer. However, severe and undesirable adverse effects remain the main complications of cancer therapy that might limit its therapeutic outcomes [31,32]. 5-FU is an effective antineoplastic agent used in the management of several solid tumors. Since 5-FU is mainly metabolized in the liver, dose-related hepatotoxicity has been associated with 5-FU therapy and may be attributed to the production of toxic metabolites [33,34]. The toxic metabolites produced by 5-FU have been blamed as being responsible for hepatic injury resulting in severe hepatotoxicity [35]. Despite the recognized risk, there are few reports examining the liver toxicity caused by 5-FU, and the underlying mechanisms remain poorly understood. Identifying these mechanisms is a critical first step in mitigating 5-FU-induced hepatic injury and developing effective hepatoprotective strategies [34,35]. 

Furthermore, much attention has been given to the inhibition of oxidative stress- and inflammatory-mediated apoptosis to mitigate liver injury induced by different toxicants [36,37,38]. It has been documented that thymol offers chemopreventive activity through its antioxidant [15], anti-inflammatory [13], and immunomodulatory mechanisms [14,39]. Therefore, the present study aimed to investigate the possible hepatoprotective effect of thymol against 5-FU-induced liver injury as well as the mechanisms of protection that might underlie it, particularly the oxidative- and inflammatory-mediated apoptosis. 

Hepatotoxicity is becoming a serious cause of morbidity and mortality. To induce hepatotoxicity in our model, two doses of 5-FU were injected consecutively and hepatotoxicity was manifested by a moderate significant elevation in liver enzymes, including AST, ALT, ALP, and LDH, compared with the control group. In addition, this elevation was found to be accompanied by marked inflammatory cell infiltration along with pyknotic degeneration in hepatocytes. This elevation in AST and ALT was probably due to the damage of liver biological membranes and endothelial linings resulting from the accumulation of oxidation products and ROS in the liver tissue. Another key hepatic marker enzyme is ALP, which is associated with membrane lipids in canalicular ducts. An elevation of ALP activity in the serum occurs when ALT and AST infiltrate the hepatocytes, thus confirming the occurrence of liver damage and indicating a biliary flow disturbance [40]. In general, these liver enzymes are considered the most significant biological markers of cellular damage and toxicity [2,41], and up to 65% of patients treated with 5-FU were known to have clinically higher levels of these liver enzymes [2,42]. Our results confirmed the previous studies, which reported that 5-FU caused elevated liver enzymes and histopathological changes in liver tissues [1,5,43]. On the other hand, thymol treatment rescued the hepatocellular damage as evidenced by the improvement in serum liver enzymes, which was associated with the considerable recovery of liver histological changes in rats who received thymol in a dose of 120 mg/kg. However, liver damage remained after thymol (60 mg/kg) treatment, including the congestion of hepatic sinusoids together with alternating intact or degenerated pyknotic hepatocytes, and was consistent with a previous study. Moreover, the dose of 120 mg/kg showed considerable improvements in the histopathological examination compared with the 5-FU group. 

It is well established that oxidative stress plays a significant role in 5-FU-induced toxicity, primarily through the involvement of reactive oxygen species (ROS) [4,44]. A study by Zeng and coworkers reported that 5-FU produces an excessive amount of ROS by damaging mitochondria morphology, resulting in hepatic dysfunction. These ROS caused lipid peroxidation and a perturbation of antioxidant balance, resulting in cell dysfunction and cell apoptosis [36]. Our findings suggest that 5-FU aggravated the oxidative stress in the liver specimens through a massive production of ROS that was confirmed by a significant increase in lipid peroxidation and depletion of enzymatic antioxidant, such as SOD activity, and non-enzymatic antioxidants, such as GSH content content in the liver. Additionally, dramatic biochemical and histological alterations in liver function and structure were associated with a marked elevation of liver lipid peroxidation and a significant decrease in antioxidants (GSH and SOD). Several previous studies demonstrated the same findings that affirmed the possible mechanism of antioxidants in mitigating 5-FU-induced liver injury [4,36]. Furthermore, thymol in either dose significantly decreased the formation of lipid peroxides, possibly by boosting the antioxidant machinery such as GSH and SOD, preserving them to normal tissue levels. This effect of thymol has been previously demonstrated against doxorubicin-mediated hepatotoxicity via the modulation of oxidative stress because of the thymol phenolic hydroxyl structure that confers the inherent antioxidant activity [18,45]. Thyme oil and thymol act as scavengers for ROS, providing substantial defense against ROS [46]. 

Another pathway that contributes to the 5-FU cytotoxicity is apoptosis, mediated by oxidative damage. Apoptosis is closely related to oxidative stress and mitigating the oxidative stress can alleviate apoptosis [36]. ROS overproduction results in potential damage to various macromolecules, and when there is insufficient protection from high ROS, cells may not survive, and the induction of apoptosis becomes inevitable [47,48,49,50]. Studies have demonstrated that 5-FU administration would cause portal apoptosis through the induction of endoplasmic reticulum stress-related genes and atrophic mitochondria [36,51]. In our experiment, we also observed that following 5-FU treatment, the hepatic expression of the apoptotic enzyme, caspase-3, significantly increased, and the anti-apoptotic protein, Bcl-2, expression was dramatically downregulated together with increased hepatic Bax protein expression, which explains the upregulation of the Bax/Bcl2 ratio in the 5-FU group compared with the control group. However, both Bax and Bcl2 proteins help suppress apoptosis in carcinogenesis rather than stimulate cell proliferation [52]. Our findings were confirmed before by the previously mentioned study [36] and were coincident with the 5-FU-induced reduction in the enzyme activity of SOD and GSH, which are essential indicators to assess the capacity to resist oxidation [50]. Accordingly, we suggested that 5-FU increased ROS production, leading to lipid peroxidation and the reduction of antioxidant enzymes, which might lead to DNA damage and promote cell death. Furthermore, our study also demonstrated that thymol significantly counteracted the 5-FU-induced hepatocyte cell apoptosis by the upregulation of Bcl2 protein expression and corrected both the Bax/Bcl2 ratio and hepatic caspase-3 expression compared with the 5-FU group. Consistently, thymol was known to reduce apoptosis by increasing Bcl-2 expression and decreasing Bax expression in different experimental models, including hepatotoxicity [24,53].

Besides Bax and Bcl-2 hepatic expression, Poly (ADP-ribose) polymerase (PARP) is a key marker involved in liver cell death. PARPs are nuclear enzymes involved in multiple biological processes, including the regulation of oxidative stress, mitochondrial function, inflammatory responses, and DNA repair processes. PARP activity increases after DNA damage induced by ROS to transfer the ADP-ribose moiety of NAD+ to form the poly(ADP-ribose) polymers. This step might make changes in the function of many enzymes and structural proteins and initiate caspase-independent cell death [54,55,56]. During apoptosis, PARP is cleaved into fragments of 89 and 24 kDa. This caspase-mediated cleavage has become a useful hallmark of apoptosis [57]. Recently, PARP activation was demonstrated in the livers of subjects with hepatitis B-mediated cirrhosis or excessive alcohol consumption, and studies reported that the inhibition of PARP has exerted a significant hepatoprotective effect via improving the inflammatory and oxidative stress-related changes induced by alcohol consumption [56,58]. 

Therefore, in our study, we investigated the cleaved PARP (89 kDa) hepatic expression in all groups, and we found a marked elevation of cleaved PARP protein expression following 5-FU administration, confirming the hepatocellular cell death. However, thymol treatment in either dose decreased the cleaved PARP protein expression, and this finding was previously reported for thymol in experimental models of lipopolysaccharides-induced nephrotoxicity [59]. We suggested that thymol might have an antiapoptotic effect driven by its inhibition of PARP signaling-mediated apoptosis.

Furthermore, mitogen-activated protein (MAP) kinases have been implicated in both cell survival and cell death. The alteration of the extracellular signal-regulated kinases (ERK1/2) pathway, which influences cell proliferation, survival, and homeostasis, has been associated with various disorders, including alcoholic liver disease [60,61]. The activation of p44/42 MAP kinase has been shown to protect against cell death [62,63]. For instance, neurotrophins exerted neuroprotection through the activation of p44/42 MAP kinase in hippocampal neuronal cells [64,65]. Our current study found that 5-FU downregulated the p44/42 MAP kinase phosphorylation, which was coincident with others reporting that ethanol-induced abnormal hepatic methionine metabolism is specifically associated with the inhibition of ERK1/2; the protective agent, betaine, improved this insult by alleviating the ERK1/2 inhibition [66]. 

In this context, one of the signaling pathways incorporated in inflammation, oxidative stress, and apoptosis is the Akt/GSK-3β signaling pathway [67]. It is known that the Akt/GSK-3β signaling pathway can be activated by Akt phosphorylation upstream and is closely linked to the PI3K/Akt pathway. In addition, a study suggested that sulforaphane inhibits neuroinflammation by modulating the PI3K/Akt/GSK-3β pathway in the rat hippocampus [68,69]. Moreover, it was documented that 5-FU inhibited cell growth and induced apoptosis by targeting Akt/GSK-3β signaling [70]. Therefore, we decided to assess the critical Akt/GSK-3β signaling pathway to further elucidate thymol’s protective effects in our experimental model of 5-FU-induced liver damage. The current study found that 5-FU elicited the activation of Akt/GSK-3β phosphorylation, as shown by the significant upregulation of the protein expression of hepatic p-AKT and p-GSK-3β. The phosphorylated Akt protein regulates a diversity of cell death pathways and cell-cycle transitions [71]. In this regard, 5-FU was known to induce drug resistance by activating p-Akt and decreasing chemotherapy sensitivity [72,73].

A study demonstrated that the activation of Akt usually involves phosphorylation at the site of threonine 308, known to phosphorylate GSK-3β on the site of serine 9 [74]. Reviewing the literature, we found that GSK-3β is constitutively active in the cytoplasm, and its activity is induced by being phosphorylated at tyrosine 216 and inhibited by phosphorylation at serine 9 [75]. Shinohara and coworkers suggested that increasing the phosphorylation of both the active and inhibited forms of GSK-3β in acetaminophen-induced hepatotoxicity resulted in an overall increase in GSK-3β activity. This previous study also suggested that GSK-3β, once further activated, is translocated to mitochondria [76]. Nevertheless, the dual and sometimes contradictory roles of GSK-3β in promoting both cell survival and apoptosis in different diseases have not been fully elucidated [23]. In the current study, we demonstrated that thymol treatment modulated Akt/ GSK-3β pathway signaling as shown by the upregulation of Akt phosphorylation and downregulation of GSK-3β phosphorylation. This effect coincided with thymol, which is a proven antioxidant and anti-apoptotic effect in our treated groups. In harmony with our findings, others have demonstrated that thymol reduced the GSK-3β protein expression in an experimental model of monosodium glutamate-induced attention deficit hyperactivity disorder [24]. Furthermore, a study by Wang et al. [25] demonstrated that thymol protected rats from glycerol-induced nephrotoxicity by increasing the phosphorylation of Akt signaling. Based on this study, it is suggested that the anti-inflammatory and anti-oxidative effects of thymol are potentially dependent on the activation of the PI3K/Akt pathway, but the exact mechanism needs further exploration. 

## 4. Materials and Methods

### 4.1. Materials

#### 4.1.1. Drugs and Chemicals

Thymol was obtained from extrasynthese (Z.I Lyon Nord, Lyon, France). We obtained the 5-FU (Fluorouracil Injection 50 mg/mL Amp) from EBEWE Pharma (Unterach am Attersee, Österreich). Primary and secondary antibodies were products of Abcam (Cambridge, MA, USA). Bax (ab6671), Bcl-2 (ab215715), cleaved PARP (46D11) Rabbit mAb, Akt 1 [(Ser473) (D9E) XP^®^ Rabbit mAb #4060.Cell sig.], phosphorylated-Akt1 (p-AKT1) [(Ser473) (D9E) XP^®^ Rabbit mAb #4060.Cell sig.], GSK-3α/β [(D75D3) Rabbit mAb], phosphorylated-GSK-3α/β (p-GSK-3α/β) [(Ser21/9) (D17D2) Rabbit mAb], and phosphorylated-44/42 MAP kinases [(Erk1/2) (137F5) Rabbit mAb #4695] and anti-glyceraldehydes-3-phosphate dehydrogenase (GAPDH) (ab8245) were used. Other chemicals were of the highest grade and were obtained from Sigma (St. Louis, MO, USA) and Abcam (Cambridge, MA, USA). 

#### 4.1.2. Animals

The study was carried out after approval was obtained from the ethical committee at the College of Pharmacy, King Saud University, Riyadh, Saudi Arabia (IRB number: KSU-SE-24-6). Forty male Wistar Rats (7 weeks old, 150–200 g) were supplied by the experimental Animal House, Faculty of Pharmacy, King Saud University, Saudi Arabia. The rats were housed in clean cages for a week before the experiment for acclimatization and kept under standard conditions, 25 °C and 60% humidity, and a 12 h light–dark cycle. In addition, rats were provided free access to a standard diet and water. According to the standard guidelines, standard diet pellets contain not less than 20% protein, 5% fiber, 3.5% fat, 6.5% ash, and a vitamin mixture. 

### 4.2. Methods

#### 4.2.1. Experiment Design

Rats were randomly organized into four different groups and treated for 11 days, as described in Table 3. 

#### 4.2.2. Blood and Liver Sample Collection

Four days after the last 5-FU dose (on the 11th day), animals were anesthetized using carbon dioxide and decapitated, and blood samples were collected from the trunk. The collected blood was centrifuged at 1000 rpm at 4 °C for 30 min, and sera were collected and stored at −80 °C. Lastly, the livers were dissected and rinsed with ice-cold phosphate-buffered saline [79]. 

A part of the liver was cut and kept in 4% formaldehyde for histopathological and immunohistochemical studies. The other part of the liver was snap frozen in liquid nitrogen and stored at −80 °C for Western blotting. The remaining part was homogenized, kept at −80 °C, and used for other biochemical assays. 

#### 4.2.3. Histopathological Examination 

Liver specimens were fixed in buffered formalin, processed, and stained with hematoxylin and eosin (H&E), as described previously [80]. Sections were then examined using a light microscope.

#### 4.2.4. Assessment of Serum Liver Enzymes and Oxidative Stress Markers in Liver Tissues

Reduced glutathione (GSH) [81], thiobarbituric acid reactive substances (TBARSs) [82], glutathione peroxidase (GPx), and superoxide dismutase (SOD) [83] were analyzed in liver homogenates using commercially available kits (Biodiagnostic, Cairo, Egypt), in compliance with the instructions of the manufacturer.

Liver enzymes, ALT, AST, ALP, and lactate dehydrogenase (LDH), were measured in sera using standard kits (United Diagnostics Industry, Dammam, Saudi Arabia).

#### 4.2.5. Assessment of Apoptotic Markers in Liver Tissues

The expression of caspase-3 in liver tissues was assessed using the immunohistochemistry technique. In addition, Bax and Bcl-2 protein expressions in liver specimens were evaluated by Western blot analysis. The immunohistochemistry was conducted following the standard protocol used in our lab [16] using the primary antibody against caspase-3 (ab32351; Abcam, Cambridge, UK). The scores for caspase-3 were calculated as the area % of the hepatic immunopositive expression of caspase-3, for which six random fields/section were used.

#### 4.2.6. Western Blot Analysis

The expression of Bax, Bcl2, cleaved PARP, Akt1, GSK-3α/β, and phosphorylated- 44/42 MAP kinase proteins in liver tissues was carried out using Western blot analysis as previously described [84].

#### 4.2.7. Assessing the Binding Affinities Using In Silico Modeling

Three proteins were selected for each selected target. Selected targets were caspase-3, GSK-3β, and BAX. Proteins were downloaded from the protein data bank with pdb codes pdb ID:7JL7 representing caspase-3, pdb ID:1Q5K representing GSK-3β, and pdb ID: 4S0P representing BAX target proteins. Ligand and protein structural optimizations were applied by Autodock4 with the aid of UCSF-chimera-1.17.3. Protein preparation, including calculating partial charges, 3D protonation, and strands correction, was performed, and then the protein was saved in pdbqt format. The ligand was optimized by UCSF-chimera and saved in a pdbqt format. A grid box was generated using autogrid 4 allocated at the macromolecule center. Docking was performed using AutoDock4. Results were then visualized using Maestro-2023-4 and Biovia-DS V21.1.0.20298.

### 4.3. Statistics

Data are represented as means ± standard deviation (SD). Multiple group analyses were performed using one-way ANOVA and then by the Tukey–Kramer post-hoc test. The significance level was accepted at *p* < 0.05. Statistical analyses and graphical representations were performed using GraphPad Prism software version 5 (ISI^®^ software, USA).

## 5. Conclusions

Collectively, our study elucidated the hepatoprotective mechanisms of thymol against 5-FU-induced hepatocellular injury in rat models. Thymol ameliorated liver damage via its antioxidant and anti-apoptotic effects. Additionally, the present study suggested that the modulation of the Akt/GSK-3β pathway signaling is potentially implicated in the cytoprotecting effect of thymol against 5-FU-induced hepatocellular death. Thus, our study might illustrate a new clinical benefit of thymol as a promising natural chemo-preventive agent in chemotherapy-based therapy for cancer patients.

## Figures and Tables

**Table 1:** Changes in liver enzymes and oxidative stress markers levels in 5-FU-induced hepatotoxicity in rats treated with thymol at two doses (60 and 120 mg/kg).

Groups	Liver Enzymes				Oxidative Stress Markers		
	AST (U/L)	ALT (U/L)	ALP (U/L)	LDH (U/L)	Glutathione (µg/mg Protein)	TBARSs (nmol/mg Protein)	SOD (U/mg Protein)
Control	92.49 ± 2.02	47.58 ± 3.91	15.89 ± 1.32	166.80 ± 1.92	1.70 ± 0.03	0.37 ± 0.02	3.93 ± 0.12
5-FU	131.20 ± 4.39 ^x^	89.21 ± 1.16 ^x^	37.80 ± 1.66 ^x^	330.60 ± 11.58 ^x^	0.63 ± 0.04 ^x^	2.60 ± 0.07 ^x^	0.95 ± 0.049 ^x^
5-FU + Thymol (60 mg/kg)	112.00 ± 6.88 ^x,y^	48.25 ± 2.73 ^y^	15.03 ± 0.85 ^y^	288.00 ± 11.58 ^x,y^	1.11 ± 0.04 ^x,y^	1.68 ± 0.03 ^x,y^	2.35 ± 0.08 ^x,y^
5-FU + Thymol (120 mg/kg)	84.58 ± 2.29 ^y,z^	31.60 ± 4.30 ^x,y,z^	11.35 ± 0.59 ^y^	182.00 ± 2.55 ^y,z^	1.55 ± 0.07 ^y,z^	0.67 ± 0.05 ^x,y,z^	3.52 ± 0.08 ^x,y,z^

Data are presented as mean ± S.D. (*n* = 6). x, y, or z: The 5-fluorouracil and Fu+ thymol (60 mg/kg) groups, are significantly different from the control at *p* < 0.05.

**Table 2:** Binding affinities and type of interactions of thymol with target proteins.

Protein	pdb ID	Binding Energy Kcal/mol	RMSD (Å)	Amino Acid Residues of Interaction	Types of Bonds
Caspase-3	3kjf	−4.16	1.48	PHE 250 ASN 208 PHE 250	H-donor pi-H pi-H
Gsk-3β	1Q5K	−5.28	1.08	VAL 135	H-donor
Bax	4S0P	−4.6	1.49	-	-

**Table 3:** The experimental groups and their description.

	Description
Control	Normal rats received the vehicle (0.5% DMSO in normal saline) once daily by oral gavage.
5-FU	Rats serving as the positive control group were given the vehicle orally (0.5% DMSO in normal saline) once daily and two doses of 5-FU (150 mg/kg, intraperitoneally (ip)) on days 6 and 7 to induce hepatic toxicity [15,77].
5-FU + Thymol 60	Rats were given thymol daily (60 mg/kg in 0.5% DMSO in normal saline, orally) and 5-FU (150 mg/kg, ip) on days 6 and 7 [15,78].
5-FU + Thymol 120	Rats were given thymol daily (120 mg/kg in 0.5% DMSO in normal saline, orally) and 5-FU (150 mg/kg, ip) on days 6 and 7 [15,24]

Animals’ weights were recorded on the 1st, 6th, and last day of the experiment.

## Data Availability

Data generated or analyzed during this study are included in this published article.
